# Flexibility of interval between vaccinations with AS03_A_-adjuvanted influenza A (H1N1) 2009 vaccine in adults aged 18–60 and >60 years: a randomized trial

**DOI:** 10.1186/1471-2334-12-162

**Published:** 2012-07-23

**Authors:** Xavier Duval, Adrian Caplanusi, Henri Laurichesse, Dominique Deplanque, Pierre Loulergue, Tejaswini Vaman, Odile Launay, Paul Gillard

**Affiliations:** 1 Université Paris Diderot, Sorbonne Paris Cité, Paris, UMR 738, France; 2 Inserm, Centre d’Investigation Clinique CIC 007-U738, Assistance Publique -Hopitaux de Paris (AP-HP), Hôpital Bichat, Paris, France; 3 GlaxoSmithCline Vaccines, Wavre, Belgium; 4 Inserm, CIC 501, CHU Clermont-Ferrand, Clermont-Ferrand, France; 5 Université Clermont-1, UMR1019 INRA, Clermont-Ferrand, France; 6 Université Lille-Nord de France, Lille, France; 7 Inserm CIC-CRB 9301, CHRU, Lille, France; 8 Inserm, CIC BT505, Assistance Publique -Hopitaux de Paris (AP-HP), Centre d’Investigation Clinique (CIC) de Vaccinologie Cochin Pasteur, Hôpital Cochin, Paris, France; 9 GlaxoSmithCline Vaccines, Bangalore, India; 10 Université Paris Descartes, Paris, France; 11 Centre d’investigation Clinique, Hôpital Bichat Claude Bernard, 46 rue Henri Huchard, Paris, 75018, France

## Abstract

**Background:**

Flexibility of vaccination schedule and lower antigen content can facilitate pandemic vaccine coverage. We assessed the immune response and safety of AS03-adjuvanted A/California/7/2009 H1N1 pandemic vaccine containing half of the registered adult haemagglutinin (HA) antigen content, administered as a two-dose schedule at intervals of 21 days or 6 months in both young and elderly adults.

**Methods:**

In this open-label randomized trial, healthy adults aged 18–60 years (N = 163) and >60 years (N = 143) received AS03_A_-adjuvanted A/California/7/2009 H1N1 vaccine containing 1.9 μg HA on Day 0. A second dose was given on Day 21 (n = 177) or Day 182 (n = 106). Haemagglutination-inhibition (HI) antibody responses were analyzed on Days 0, 21, 42, 182, 364 and additionally on Day 203 for subjects vaccinated on Day 182. Solicited and unsolicited adverse events were recorded.

**Results:**

The HI antibody response in both age strata 21 days after the first dose met and exceeded all regulatory acceptance criteria although the results suggested a lower response in the older age stratum (geometric mean titres [GMTs] for HI antibodies of 420.5 for subjects aged 18–60 years and 174.4 for those >60 years). A second dose of AS03_A_ adjuvanted A/H1N1/2009 vaccine induced a further increase in antibody titres and the response was similar whether the second dose was administered at 21 days (GMTs of 771.8 for 18–60 years and 400.9 for >60 years) or 6 months (GMTs of 708.3 for 18–60 years and 512.1 for >60 years) following the first dose. Seroprotection rates remained high at 6 months after one dose or two doses while at 12 months rates tended to be higher for the 6 month interval schedule (93.3% for 18–60 years and 80.4% for >60 years) than the 21 day schedule (82.3% for 18–60 years and 50.0% for >60 years). Reactogenicity/safety profiles were similar for both schedules, there was no evidence of an increase in reactogenicity following the second dose.

**Conclusions:**

The results indicate that flexibility in the dosing interval for AS03_A_ adjuvanted vaccine may be possible. Such flexibility could help to reduce the logistic stress on delivery of pandemic vaccination programmes.

**Trial registration:**

ClinicalTrials.gov, NCT00975884

## Background

Influenza A viruses are responsible for annual seasonal epidemics in humans when a limited number of mutations occur (drift) and for influenza pandemics when, following a substantial number of mutations or gene segment reassortment (shift), a new variant of influenza virus emerges against which humans have poor or no existing immunity. The highly pathogenic avian influenza A H5N1 strain has been circulating in several countries during the last few years and, although not readily transmissible, new drift variants are continuously emerging which could acquire more efficient human to human transmission [1].

Vaccination remains the most effective method for preventing influenza infection [2] despite the complicated logistics of large scale pandemic vaccination campaigns. Two particular challenges for pandemic vaccines are to achieve immunogenicity with the lowest antigen content, given the limited global influenza antigen manufacturing capacity [3] and to maximise the cross-reactive potential of pandemic antigen against possible drift strains.

During development of vaccines against avian H5N1, adjuvant technology was successfully applied to address these challenges. Inclusion of the α-tocopherol oil-in-water emulsion based Adjuvant System AS03 enhanced the immunogenicity of H5N1 vaccines thereby reducing the amount of HA antigen required to 3.75 μg per dose of vaccine [4]. AS03 also stimulated cross-immunity against drifted H5N1 strains [4,5] and induced protection against heterologous lethal H5N1 challenge in ferrets [6]. As AS03 adjuvanted H5N1 formulations demonstrated a clinically acceptable safety profile [7,8], the AS03 Adjuvant System was incorporated in the pandemic vaccine produced in response to the emergence of A/H1N1/2009 virus. AS03-adjuvanted A/H1N1/2009 vaccine was authorised for use at an HA antigen dose of 3.75 μg for adults and 1.9 μg for children [9,10] in mass vaccination campaigns in many countries.

In order to increase vaccine availability, an expansion of manufacturing capacity and/or further reductions in vaccine antigen content may be required. Another issue was the logistics of administration of two doses of vaccine given 21 days apart which was the initial schedule recommended based on experience with H5N1 vaccine. Flexibility in this schedule, permitting an increase in the dosing interval, could help to increase vaccine coverage during the initial phase of a pandemic when vaccine supplies may be limited. An increase in dosing interval could potentially promote persistence of antibodies over a longer period which may be an advantage during prolonged circulation of a pandemic virus. A recent study showed that one dose of AS03-adjuvanted H5N1 vaccine, followed by a single-adjuvanted heterologous booster 12 months later elicited immune responses that met all US and European criteria for both strains after the booster dose [11].

Immunogenicity data has indicated that unlike H5N1, one dose of A/H1N1/2009 vaccine is sufficient to elicit a satisfactory immune response in most age groups [9,10,12-16]. However, planning for future pandemics still needs to anticipate the possibility of a two dose schedule as the number of doses required may depend on the nature of the pandemic strain and will not be known until the immunogenicity profile has been established.

We report on the immune response and safety in both young and elderly adults of AS03 adjuvanted A/California/7/2009 H1N1 vaccine containing half of the antigen content per dose (1.9 μg instead of 3.75 μg HA) registered for adults but maintaining the same level of adjuvant as in the registered adult dose. In addition we assessed the flexibility of the administration schedule at 21 days or 6 months interval. An interval of 6 months was selected based on previous experience with AS03 adjuvanted H5N1 vaccine [17].

## Methods

### Study design and participants

This was a phase III open, randomized trial with two groups conducted in five centres in France from September 2009 to April 2010. The primary objective was to demonstrate that vaccination with one dose of A/H1N1/2009 vaccine containing 1.9 μg of HA adjuvanted with AS03_A_ results in a haemagglutination-inhibition (HI) immune response that meets or exceeds European Medicines Agency (EMA) Committee for Medicinal Products for Human Use (CHMP) criteria for pandemic vaccines 21 days post vaccination [18]. Secondary objectives included the assessment of immunogenicity following administration of a second dose at Day 21 or Day 182, assessment of persistence of the antibody response at Day 182 and Day 364, and assessment of reactogenicity and safety.

Eligible participants enrolled by the responsible on-site personnel were clinically healthy non-pregnant adults over 18 years of age at the time of the first vaccination who provided written informed consent. Subjects with a clinical history suggestive of an influenza infection within 6 months preceding the study start were excluded. The enrolment was age-stratified, with two age strata (18–60 years and >60 years, in 1:1 ratio) with a minimum of 40% of subjects aged 18–40 years and a minimum of 40% of subjects aged 41–60 years, in the 18–60 years stratum; and a minimum of 40% of subjects aged 61–70 years and a minimum of 20% of subjects aged above 70, in the >60 years stratum.

In the initial study design, all subjects were to be vaccinated on Day 0 and Day 21, however, the protocol was amended soon after the enrolment of the first subjects (and before Day 21 was reached for these subjects) to include a Day 0 and Day 182 immunization schedule. This amendment provided the opportunity to investigate the immune response 6 months after a single A/H1N1/2009 vaccine injection and to assess the flexibility of the vaccination schedule. The list of vaccinated subject numbers which was tracked using the central randomisation system on Internet (SBIR) and was randomised (1:1) by the sponsor using SAS version 9.2 to receive a second dose at either Day 21 (Group A) or Day 182 (Group B) taking into account the minimisation procedure for centre and age. At Day 21, the subjects were asked by the responsible on-site personnel to consent to either receive a second vaccination at Day 21 as initially planned or to be randomly assigned to a Day 21 or Day 182 second dose administration.

The protocol, its amendments and other relevant study documentation were approved by the appropriate Ethics Committee (CPP 1, Ile de France) and the study was conducted in accordance with good clinical practice guidelines, the Declaration of Helsinki and all applicable regulatory requirements.

### Study vaccine

The monovalent influenza A/H1N1/2009 inactivated, split-virion vaccine was manufactured by GlaxoSmithKline (GSK) Vaccines from a vaccine seed virus prepared from the reassortant virus NYMC X-179A (New York Medical College, New York) generated from the A/California/7/2009 strain as recommended by the World Health Organization (WHO) [19]. The seed virus was propagated in embryonated hen eggs and the vaccine was produced using the licensed manufacturing process for *Pandemrix*™ (a trade mark of the GlaxoSmithKline group of companies). AS03_A_ is an Adjuvant System containing α-tocopherol and squalene in an oil-in-water emulsion (11.86 mg α-tocopherol) manufactured by GSK Vaccines. The study vaccine was formulated to contain 1.9 μg of HA antigen and AS03_A_ [note that the standard dose registered for adults contains 3.75 μg of HA and AS03_A_ while the paediatric dose contains 1.9 μg of HA and AS03_B_ (5.93 mg α-tocopherol)].

### Procedures

A dose of AS03_A_-adjuvanted vaccine (0.38 ml injection volume in this trial) was administered intramuscularly in the deltoid region on Day 0 and either on Day 21 or Day 182 (according to subject consent to be randomly assigned or not on Day 21 to one of the two schedules). Blood samples were collected on Day 0 (prior to vaccination), Day 21, Day 42 and Day 182, Day 364 and additionally for Group B only, on Day 203. All samples were tested under blind conditions n a validated micro-titre HI test using chicken erythrocytes as previously described [20] with the A/California/7/2009 strain used as antigen.

Data on solicited symptoms experienced within the first 7 days following each vaccination were recorded on diary cards by participants as previously described [21]. Data were also collected on the occurrence of any unsolicited adverse events occurring within 21 days after the first vaccination and either 63 days after the second vaccination (for the 21 days interval schedule group) or 30 days after the second vaccination (for the 6 months interval schedule group). The intensities of symptoms and adverse events were recorded according to a standard three-grade scale as previously described [21]. An assessment of causality was made by the investigator for solicited systemic symptoms and unsolicited adverse events. Data on serious adverse events, adverse events of specific interest (AESIs) also called potential Immune-Mediated Diseases (pIMDs) and adverse events of special interest (convulsion and anaphylaxis) are planned to be collected up to Day 546. This paper presents data up until Day 364.

### Statistical analysis

The analysis was descriptive. The immune response at Day 21 addressing the primary objective was analysed according to the standard EMA CHMP HI endpoints [with 95% confidence intervals (CI)] used for evaluation of influenza vaccines [18]. The sample size of 135 evaluable subjects per age strata (270 in total) was calculated to give a power of at least 95% to fulfil the CHMP criteria for these endpoints [18] following one dose of vaccine. The immune response after the second dose was also assessed for the same endpoints.

The safety endpoints (percentage of subjects and 95% CI) were solicited adverse events and spontaneously reported adverse events. All serious adverse events occurring up to Day 364 were described. The statistical analysis was performed using SAS Version 9.2.

Safety was assessed in the Total Vaccinated Cohort including all subjects who received at least one dose of vaccine. Immunogenicity was assessed in the per protocol population including subjects without protocol violation and with serological data at each immunogenicity time point (Day 21, Day 42, Day 182, Day 203 (Group B), and Day 364).

## Results

### Study population

**Figure 1:**
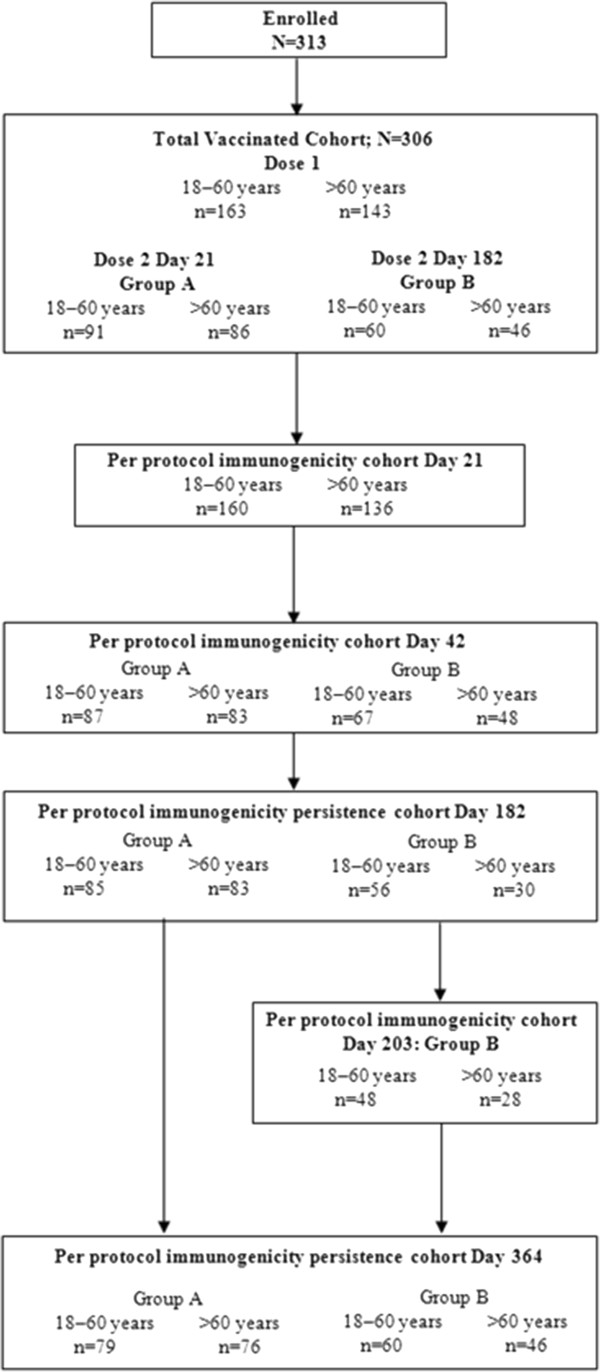
Trial Profile.

A total of 306 subjects were vaccinated with AS03_A_-adjuvanted A/H1N1/2009 vaccine on Day 0. Of these, 177 subjects received a 2^nd^ dose on Day 21 (group A) and 106 subjects received a 2^nd^ dose on Day 182 (group B). The stratification according to age led to the initial vaccination of 163 subjects aged 18 to 60 years and of 143 subjects aged over 60 years. Figure 1 shows the trial profile and Additional file 1: Table S1 provides the cohorts and rationale for exclusions from the immunogenicity assessment at each timepoint. The demographic profiles for the per-protocol analysis of immunogenicity for each group were similar with respect to age at first dose vaccination (38.9 and 41.2 years and 66.6 and 66.3 years for the two age strata for groups A and B, respectively) gender distribution (approximately 1:1) and geographic ancestry (mainly white Caucasian).

### Immune response

**Figure 2:**
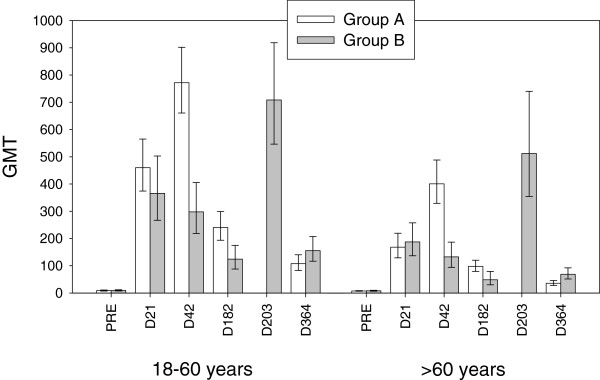
**Geometric mean titres (with 95% confidence intervals) for haemagglutination inhibition antibodies against A/California/7/2009 in adults aged 18 to 60 years and in adults >60 years (per-protocol immunogenicity cohorts).** Group A = Vaccination on Day 0 and Day 21; Group B = Vaccination on Day 0 and Day 182.

The HI antibody responses following both schedules in adults aged 18 to 60 years and above 60 years are presented in Table 1 and Figure 2.

**Table 1:** Immune response for haemagglutination-inhibition (HI) antibodies against A/California/7/2009 H1N1 in adults aged 18 to 60 years and in adults >60 years (per-protocol immunogenicity cohorts)

	**N**	**Seroprotection rate (titre ≥ 1:40) % (95% CI)**	Seroconversion rate* % (95% CI)	Geometric Mean Fold Rise** (95% CI)	**N**	**Seroprotection rate (titre ≥ 1:40) % (95% CI)**	Seroconversion rate* % (95% CI)	Geometric Mean Fold Rise** (95% CI)
		**Vaccinated on Day 0 (subjects from Groups A and B pooled) 18**–**60 years**				**Vaccinated on Day 0 (subjects from Groups A and B pooled) 18>60 years**		
**Day 0 (Prevaccination)**	160	**14.4**	-	-	136	**5.1**	-	-
		(9.3-20.8)				(2.1-10.3)		
**Day 21**	160	**97.5**	**96.3**	**45.0**	136	**91.9**	**89.0**	**23.4**
		(93.7-99.3)	(92.0-98.6)	(37.3-54.5)		(86.0-95.9)	(82.5-93.7)	(19.1-28.7)
	**Group A Vaccinated on Day 0 and Day 21 18**–**60 years**				**Group A Vaccinated on Day 0 and Day 21 >60 years**			
**Day 0 (Prevaccination)**	87	**12.6**	-	-	83	**7.2**	-	-
		(6.5-21.5)		-		(2.7-15.1)		
**Day 21**	87	**100**	**100**	**51.6**	83	**90.4**	**89.2**	**23.0**
		(95.8-100)	(95.8-100)	(40.7-65.5)		(81.9-95.7)	(80.4-94.9)	(17.7-29.9)
**Day 42**	87	**100**	**98.9**	**86.7**	83	**100**	**98.8**	**54.9**
		(95.8-100)	(93.8-100)	(68.6-109.5)		(95.7-100)	(93.5-100)	(43.4-69.3)
**Day 182**	85	**97.6**	**92.9**	**26.6**	83	**90.4**	**86.7**	**13.4**
		(91.8-99.7)	(85.3-97.4)	(20.8-34.0)		(81.9-95.7)	(77.5-93.2)	(10.9-16.5)
**Day 203**		-	-	-		-	-	-
**Day 364**	79	**82.3**	**75.9**	**11.0**	76	**50.0**	**40.8**	**4.8**
		(72.1-90.0)	(65.0-84.9)	(9.2-15.5)		(38.3-61.7)	(29.6-52.7)	(3.8-6.1)
	**Group B Vaccinated on Day 0 and Day 182 18**–**60 years**				**Group B Vaccinated on Day 0 and Day 182 >60 years**			
**Day 0 (Prevaccination**	67	**14.9** (7.4-25.7)	-	-	48	**2.1** (0.1-11.1)	-	-
**Day 21**	67	**94.0**	**92.5**	**39.2**	48	**95.8**	**89.6**	**23.3**
		(85.4-98.3)	(83.4-97.5)	(28.3-54.2)		(85.7-99.5)	(77.3-96.5)	(16.6-32.7)
**Day 42**	67	**95.5**	**91.0**	**31.9**	48	**89.6**	**81.3**	**16.5**
		(87.5-99.1)	(81.5-96.6)	(23.4-43.4)		(77.3-96.5)	(67.4-91.1)	(11.8-23.0)
**Day 182**	56	**85.7**	**83.9**	**14.6**	30	**63.3**	**56.7**	**6.1**
		(73.8-93.6)	(71.7-92.4)	(10.8-19.7)		(43.9-80.1)	(37.4-74.5)	(3.9-9.4)
**Day 203**	48	**100**	**100**	**79.0**	28	**100**	**100**	**65.7**
		(92.6-100)	(92.6-100)	(58.0-107.7)		(87.7-100)	(87.7-100)	(41.4-104.1)
**Day 364**	60	**93.3**	**90.0**	**17.3**	46	**80.4**	**73.9**	**8.3**
		(83.8-98.2)	(79.5-96.2)	(13.0-23.0)		(66.1-90.6)	(58.9-85.7)	(6.0-11.3)

*Seroconversion rate for haemagglutination-inhibition antibody response is defined as the percentage of vaccinees who have a prevaccination titre <1:10 and a post-vaccination titre ≥1:40, or a significant increase in antibody titre defined as the percentage of vaccinees who have a pre-vaccination titre ≥ 1:10 and at least a fourfold increase in post-vaccination titre. **Geometric mean fold rise is defined as geometric mean of the within-subject ratios of the post-vaccination reciprocal haemagglutination-inhibition titre to the Day 0 reciprocal agglutination-inhibition titre.

European Medicines Agency Committee for Medicinal Products for Human Use (CHMP) criteria for haemagglutination-inhibition antibody response in 18–60 year olds are: seroprotection rate >70%, seroconversion rate >40%, and geometric mean fold rise >2·5 and in adults >60 years are seroprotection rate >60%, seroconversion rate >30%, and geometric mean fold rise >2·0.

United States Center for Biologics Evaluation and Research (CBER) criteria for hemagglutination-inhibition antibody response in adults <65 years are: lower limit (LL) of 95% CI for seroprotection ≥70% and LL of 95% CI for seroconversion ≥40% and in adults ≥65 years are LL of 95% CI for seroprotection ≥60% and LL of 95% CI for seroconversion ≥30%.

Prior to vaccination the percentages of subjects with pre-existing HI antibody levels ≥1:40 (considered to be seroprotective) were 14.4% for subjects aged <18–60 years and 5.1% for subjects aged >60 years older adults and pre-vaccination Geometric Mean Titres (GMTs) in seropositive subjects were low in both age groups (28.1 and 16.3).

The HI response in both age strata on Day 21 following the first dose of vaccine met and exceeded all European CHMP regulatory acceptance criteria (as well as CBER criteria) for influenza vaccines and so the primary objective of the study was achieved. The results suggested a lower immune response in the older age stratum (GMTs of 459.8 for 18–60 year olds and 168.2 for above 60 year olds for pooled groups). Further stratification by age suggested slightly lower GMTs with advancing age at all time-points (data not shown).

In group A, a second dose administered at Day 21 to 177 subjects induced a further increase in HI GMTs at Day 42 in both age strata (from 459.8 after the first vaccination to 771.8 for the 18–60 years group and from 168.2 after the first vaccination to 400.9 for the >60 years group). In group B, at 42 days after a single vaccine dose, GMT values had decreased only marginally compared to the previous time point.

HI antibodies still persisted at 6 months (Day 182) with GMTs of 240.5 (18 to 60 years) and 97.8 (>60 years) in the group that had received two doses (group A) and 124.1 (18–60 years) and 48.6 (>60 years) in the group that had only received a single dose up to that time-point (group B). Despite the decline in GMTs, the percentages of subjects with putatively seroprotective antibody levels at 6 months following administration of either one (85.7% and 63.3% of the 18–60 years and the >60 years groups, respectively) or two vaccine doses (97.6% and 90.4% of the 18–60 years and >60 years groups, respectively) still complied with European regulatory criteria (also for seroconversion rates and geometric mean fold rises) and except for the older age stratum who received only one dose, still also complied with US regulatory criteria.

A second dose administered at 6 months (Day 182) interval to 106 subjects induced a further increase of GMTs 21 days later (on Day 203) in both age strata (708.3 for 18–60 years and 512.1 for >60 years). The GMTs induced by a second dose were similar whether administered at Day 21 or at 6 months interval.

HI antibody persistence at study month 12 (Day 364) tended to be higher for the 6 month interval group B (GMTs and seroprotection rates were respectively 155.5 and 93.3% for 18–60 years and 68.8 and 80.4% for >60 years) than for the 21 day interval group A (GMTs and seroprotection rates were respectively 107.8 and 82.3% for 18–60 years and 35.8 and 50.0% for >60 years).

### Reactogenicity and safety

The incidence of solicited local (injection site) and systemic symptoms reported per group within 7 days after each vaccination are presented in Tables 2 and 3 respectively.

**Table 2:** Percentage (with 95% confidence intervals) of subjects reporting solicited local (injection site) symptoms within 7 days after the first and after the second dose of H1N1 vaccine in adults aged 18 to 60 years and in adults >60 years

**Symptom**	**Intensity**	**Group A Vaccinated on Day 0 and Day 21**		**Group B Vaccinated on Day 0 and Day 182**	
		**Dose 1% (95% CI)**			
		**18-60 years N = 92**	**>60 years N = 90**	**18-60 years N = 70**	**>60 years N = 52**
**Pain**	Any	**94.6**	**78.9**	**95.7**	**67.3**
		(87.8-98.2)	(69.0-86.8)	(88.0-99.1)	(52.9-79.7)
	Grade 3*	**0.0**	**2.2**	**0.0**	**0.0**
		(0.0-3.9)	(0.3-7.8)	(0.0-5.1)	(0.0-6.8)
**Redness**	Any	**10.9**	**8.9**	**11.4**	**1.9**
		(5.3-19.1)	(3.9-16.8)	(5.1-21.3)	(0.0-10.3)
	>100 mm	**0.0**	**0.0**	**0.0**	**0.0**
		(0.0-3.9)	(0.0-4.0)	(0.0-5.1)	(0.0-6.8)
**Swelling**	Any	**12.0**	**6.7**	**8.6**	**1.9**
		(6.1-20.4)	(2.5-13.9)	(3.2-17.7)	(0.0-10.3)
	>100 mm	**0.0**	**0.0**	**0.0**	**0.0**
		(0.0-3.9)	(0.0-4.0)	(0.0-5.1)	(0.0-6.8)
		**Dose 2% (95% CI)**			
		**18-60 years N = 91**	**>60 years N = 85**	**18-60 years N = 60**	**>60 years N = 45**
**Pain**	Any	**87.9**	**72.9**	**90.0**	**57.8**
		(79.4-93.8)	(62.2-82.0)	(79.5-96.2)	(42.2-72.3)
	Grade 3*	**2.2**	**0.0**	**5.0**	**0.0**
		(0.3-7.7)	(0.0-4.2)	(1.0-13.9)	(0.0-7.9)
**Redness**	Any	**6.6**	**12.9**	**10.0**	**6.7**
		(2.5-13.8)	(6.6-22.0)	(3.8-20.5)	(1.4-18.3)
	>100 mm	**0.0**	**0.0**	**3.3**	**2.2**
		(0.0-4.0)	(0.0-4.2)	(0.4-11.5)	(0.1-11.8)
**Swelling**	Any	**8.8**	**11.8**	**16.7**	**8.9**
		(3.9-16.6)	(5.8-20.6)	(8.3-28.5)	(2.5-21.2)
	>100 mm	**0.0**	**0.0**	**0.0**	**0.0**
		(0.0-4.0)	(0.0-4.2)	(0.0-6.0)	(0.0-7.9)

*Grade 3 was defined as significant injection site pain at rest which prevents normal activities (inability to attend/do work).

**Table 3:** Percentage of subjects (with 95% confidence intervals) reporting solicited systemic symptoms within 7 days after the first and after the second dose of H1N1 vaccine in adults aged 18 to 60 years and in adults >60 years

**Symptom**	**Intensity**	**Group A Vaccinated on Day 0 and Day 21**		**Group B Vaccinated on Day 0 and Day 182**	
		**Dose 1% (95% CI)**			
		**18-60 years N = 92**	**>60 years N = 90**	**18-60 years N = 70**	**>60 years N = 52**
**Fatigue**	Any	**43.5**	**28.9**	**38.6**	**34.6**
		(33.2-54.2)	(19.8-39.4)	(27.2-51.0)	(22.0-49.1)
	Grade 3*	**0.0**	**0.0**	**1.4**	**0.0**
		(0.0-3.9)	(0.0-4.0)	(0.0-7.7)	(0.0-6.8)
**Headache**	Any	**37.0**	**22.2**	**31.4**	**17.3**
		(27.1-47.7)	(14.1-32.2)	(20.9-43.6)	(8.2-30.3)
	Grade 3*	**0.0**	**1.1**	**1.4**	**0.0**
		(0.0-3.9)	(0.0-6.0)	(0.0-7.7)	(0.0-6.8)
**Joint pain at other location**	Any	**22.8**	**21.1**	**14.3**	**15.4**
		(14.7-32.8)	(13.2-31.0)	(7.1-24.7)	(6.9-28.1)
	Grade 3*	**0.0**	**0.0**	**0.0**	**0.0**
		(0.0-3.9)	(0.0-4.0)	(0.0-5.1)	(0.0-6.8)
**Muscle aches**	Any	**46.7**	**28.9**	**32.9**	**28.8**
		(36.3-57.4)	(19.8-39.4)	(22.1-45.1)	(17.1-43.1)
	Grade 3*	**0.0**	**0.0**	**0.0**	**1.9**
		(0.0-3.9)	(0.0-4.0)	(0.0-5.1)	(0.0-10.3)
**Shivering**	Any	**18.5**	**13.3**	**20.0**	**11.5**
		(11.1-27.9)	(7.1-22.1)	(11.4-31.3)	(4.4-23.4)
	Grade 3*	**0.0**	**1.1**	**0.0**	**0.0**
		(0.0-3.9)	(0.0-6.0)	(0.0-5.1)	(0.0-6.8)
**Sweating**	Any	**13.0**	**11.1**	**10.0**	**13.5**
		(6.9-21.7)	(5.5-19.5)	(4.1-19.5)	(5.6-25.8)
	Grade 3*	**1.1**	**0.0**	**0.0**	**0.0**
		(0.0-5.9)	(0.0-4.0)	(0.0-5.1)	(0.0-6.8)
**Fever (Axillary temp)**	Any	**1.1**	**1.1**	**2.9**	**1.9**
		(0.0-5.9)	(0.0-6.0)	(0.3-9.9)	(0.0-10.3)
	≥39.0 to ≤40°C	**0.0**	**0.0**	**0.0**	**0.0**
		(0.0-3.9)	(0.0-4.0)	(0.0-5.1)	(0.0-6.8)
		**Dose 2% (95% CI)**			
		**18-60 years N = 91**	**>60 years N = 85**	**18-60 years N = 60**	**>60 years N = 45**
**Fatigue**	Any	**53.8**	**31.8**	**51.7**	**29.5**
		(43.1-64.4)	(22.1-42.8)	(38.4-64.8)	(16.8-45.2)
	Grade 3*	**3.3**	**1.2**	**3.3**	**0.0**
		(0.7-9.3)	(0.0-6.4)	(0.4-11.5)	(0.0-8.0)
**Headache**	Any	**45.1**	**22.4**	**35.0**	**25.0**
		(34.6-55.8)	(14.0-32.7)	(23.1-48.4)	(13.2-40.3)
	Grade 3*	**1.1**	**1.2**	**5.0**	**0.0**
		(0.0-6.0)	(0.0-6.4)	(1.0-13.9)	(0.0-8.0)
**Joint pain at other location**	Any	**19.8**	**24.7**	**26.7**	**27.3**
		(12.2-29.4)	(16.0-35.3)	(16.1-39.7)	(15.0-42.8)
	Grade 3*	**0.0**	**0.0**	**0.0**	**0.0**
		(0.0-4.0)	(0.0-4.2)	(0.0-6.0)	(0.0-8.0)
**Muscle aches**	Any	**42.9**	**29.4**	**51.7**	**34.1**
		(32.5-53.7)	(20.0-40.3)	(38.4-64.8)	(20.5-49.9)
	Grade 3*	**0.0**	**0.0**	**1.7**	**0.0**
		(0.0-4.0)	(0.0-4.2)	(0.0-8.9)	(0.0-8.0)
**Shivering**	Any	**28.6**	**15.3**	**18.3**	**15.9**
		(19.6-39.0)	(8.4-24.7)	(9.5-30.4)	(6.6-30.1)
	Grade 3*	**1.1**	**1.2**	**0.0**	**0.0**
		(0.0-6.0)	(0.0-6.4)	(0.0-6.0)	(0.0-8.0)
**Sweating**	Any	**20.9**	**16.5**	**13.3**	**9.1**
		(13.1-30.7)	(9.3-26.1)	(5.9-24.6)	(2.5-21.7)
	Grade 3*	**1.1**	**0.0**	**0.0**	**0.0**
		(0.0-6.0)	(0.0-4.2)	(0.0-6.0)	(0.0-8.0)
**Fever (Axillary temp)**	Any	**6.6**	**5.9**	**1.7**	**0.0**
		(2.5-13.8)	(1.9-13.2)	(0.0-8.9)	(0.0-8.0)
	≥39.0 to ≤40°C	**0.0**	**2.4**	**0.0**	**0.0**
		(0.0-4.0)	(0.3-8.2)	(0.0-6.0)	(0.0-8.0)

*Grade 3 events were defined as events that prevent normal everyday activities (inability to attend/do work), or that require intervention of a physician/healthcare provider.

The profile and frequencies of solicited symptoms were similar in both study groups. No difference in profile or increase in the frequencies of solicited symptoms could be distinguished with the administration of a second dose in either vaccination schedule. The observed incidences of certain solicited symptoms were slightly numerically higher in the subjects aged 18–60 years.

Pain at the injection site was the most common solicited symptom reported following vaccination in both younger adults (94.6% and 95.7% after a first dose and 87.9% and 90% after a second dose in group A (Day 0 - Day 21 vaccinations) and group B (Day 0 - Day 182 vaccinations, respectively) and among adults aged >60 years (78.9% and 67.3% after a first dose and 72.9% and 57.8% after a second dose in groups A and B, respectively). Most injection site related symptoms were mild to moderate with a maximum of three subjects per age stratum reporting grade 3 pain or redness or swelling greater than 100 mm after any vaccination. The most frequently reported solicited systemic symptoms were fatigue (maximum 53.8% after a second dose at Day 21, 18–60 years stratum), muscle aches (maximum 51.7% after a second dose at Day 182, 18–60 years stratum) and headache (maximum 45.1% after a second dose at Day 21, 18–60 years stratum). Grade 3 systemic symptoms were rare (0 to 4 subjects for any one of these symptoms after every dose).

In group A the percentage of subjects reporting unsolicited adverse events (data not shown) within 21 days after the first vaccination and 63 days after the second vaccination was 55.9% of the 18–60 year age group (12.9% of these events were considered as related to vaccination) and 58.2% of the >60 year age group (16.5% considered as related to vaccination). In group B the percentage of subjects reporting unsolicited adverse events (data not shown) within 21 days after the first vaccination and 30 days after the second vaccination was 50.0% in the 18–60 year age group (18.6% considered as related to vaccination) and 30.8% in the >60 year age group (9.6% considered as related to vaccination). The most frequently reported adverse events were rhinitis, nasopharyngitis and bronchitis. Grade 3 adverse events were reported by 10.8% of subjects aged 18–60 years (none considered as related to vaccination) and 9.9% of subjects aged >60 years (1.1% considered as related to vaccination) in group A and by 8.6% of subjects aged 18–60 years (4.3% considered as related to vaccination) and 3.8% of subjects aged >60 years (1.9% considered as related to vaccination) in group B.

Over the Day 0 to Day 364 study period reported here, nineteen serious adverse events were reported of which two were considered by the investigator as related to the vaccination. A 63 year-old female experienced hepatic enzyme increase at 21 days after the first vaccination; this was resolved on Day 42. The second event was a Herpes zoster infection reported 18 days following the second dose and resolved 22 days later.

There was one withdrawal from the study due to a non-serious adverse event. Up to Day 364, two pIMDs were reported (ankylosing spondylitis 157 days after the 2^nd^ dose and multiple sclerosis 37 days after the 2^nd^ dose). Neither event was considered by the investigator to be related to vaccination. There were two events of special interest reported: multiple sclerosis and urticaria. The urticaria event, reported on day 1 after the 1^st^ dose, was considered by the investigator to be related to vaccination and resolved 15 days later.

## Discussion

The results of this study indicate that for adults aged 18 to 60 years and elderly adults over 60 years the dosing interval between vaccinations with AS03_A_-adjuvanted influenza A (H1N1) 2009 vaccine could be flexible.

The genetic variability of influenza A viruses means that the characteristics of the next pandemic influenza strain are not predictable; it could be a return of H2N2, a variant of the swine-origin A/H1N1/2009 strain or currently circulating H3N2, a more transmissible variant of the avian-origin H5, H9, or H7 subtypes or a completely novel strain. It is important therefore to build up a knowledge base from data generated with A/H1N1/2009, H5N1 and other subtype vaccines which could be potentially relevant to development of new pandemic vaccines.

Even though a single dose of A/H1N1/2009 vaccine appeared to be sufficient to fulfil regulatory criteria for 2009 pandemic influenza vaccine [9,10,12-16], this may not be the case for future pandemic influenza strains depending on how antigenically and genetically distinct they are from strains which have previously circulated in humans. It has already been shown that for the avian H5N1 influenza strain, two doses of vaccine are required to elicit a satisfactory response [4], although a recent study showed that two heterologous doses given 12 months apart elicited immune responses that met all US and European criteria for both H5N1 vaccine strains after the booster dose [11]. This observation concurs with data generated with AS03_A_ adjuvanted A/Vietnam/1194/2004 (H5N1) vaccine which showed that two doses given 6 months apart achieved equivalent seroprotection after the second vaccination when compared to two doses given 21 days apart [17]. Furthermore, when immunity against the heterologous A/Indonesia/05/2005 H5N1 strain was measured, the group vaccinated at 0 and 6 months achieved an even higher cross-reactive response after the last vaccination, when compared with the group vaccinated at 0 and 21 days [17]. In our study the cross-reactive responses were not investigated for the two schedules as no antigenically drifted strain from the A/H1N1/2009 strain has yet been isolated. In terms of antibody persistence although at 12 months following the first dose, the seroprotection rates tended to be higher for the 0 and 6 months schedule, about 80% of 18–60 year olds and 50% of the over 60 year olds vaccinated according to the 0 and 21 day schedule still had seroprotective antibody levels at 12 months.

Previous studies have shown that when combined with AS03_A_ adjuvant, one dose of A/H1N1/2009 vaccine containing 3.75 μg of HA antigen is sufficient to induce a response meeting regulatory criteria for influenza vaccines in adults at 21 days following vaccination [10,22]. In this study the immune response induced by one dose of AS03_A_ adjuvanted A/H1N1/2009 vaccine containing the lower antigen content of 1.9 μg HA has met and exceeded all European and US regulatory criteria in both younger (18–60 years) and older (above 60 years) adults. Furthermore at six months following administration of one vaccine dose containing 1.9 μg HA, even though antibody levels had declined, seroprotective rates still complied with European criteria and also with US criteria for younger adults. These data suggested that use of AS03_A_ adjuvant may allow the vaccine antigen content to be further reduced below 3.75 μg HA. Although this observation is specific to A/H1N1/2009 and would also have to be ascertained for other strains such as H5N1, it merits further investigation in the context of potential antigen shortage. Further studies would however need to include a control group administered with 3.75 μg HA to ascertain the impact on the magnitude of the antibody response and persistence, particularly in older adults where the response tended to be lower than in younger adults. Older adults also tend to have weaker immune responses to non-adjuvanted seasonal influenza vaccines than younger adults and this has been attributed to a decline in immune function with increasing age [23-25]. Inclusion of oil in water based emulsion adjuvants however has been shown to improve the immunogenicity of seasonal [26], H5N1 [27] and A/H1N1/2009 influenza vaccines [30] in the elderly.

A limitation of the study was that the population in Group B beyond Day 21 was below the pre-specified 135 subjects needed to demonstrate that immunogenicity fulfilled the CHMP criteria with 95% power. Although the immunogenicity endpoints fulfilled the licensure criteria in Group B at Day 182 and Day 203, it should be noted that the per protocol populations at these time points comprised 86 and 76 subjects, respectively. Although subjects with a medical history suggesting influenza during the six months prior to the study were excluded, seroprotection rates of 14% for adults aged 18 to 60 years and 5% for adults aged over 60 years were recorded prior to vaccination. The presence of baseline antibodies against A/H1N1/2009 has also been documented in other studies [12-14,21]. As Hancock et al. [28] concluded that recent seasonal influenza vaccines induced little or no cross-reactive antibody responses to A/H1N1/2009, these baseline antibodies might be due to asymptomatic A/H1N1/2009 infections prior to the study start (September 2009). It has also been speculated that some older adults may have been exposed to strains closely related to A/H1N1/2009 which were in circulation over 60 years ago [28,29].

The AS03 Adjuvant System has previously been administered with H5N1 vaccines in a two-dose schedule to a large number of adults in clinical studies [7,8] and more recently with A/California/7/2009 H1N1 vaccine as a single-dose schedule in mass vaccination campaigns in many countries. Consistent with other published clinical studies on AS03 adjuvanted A/California/7/2009 H1N1 [10,21,22] or H5N1 vaccines [7,8] the most common solicited symptoms following vaccination in this present study in adults aged 18 to 60 years and over 60 years were injection site pain, fatigue, headache, and muscle aches which were mainly mild to moderate in nature.

Reactogenicity was within the same range after the first and after the second vaccine dose whether or not they were 21 days or 6 months apart and also for both study groups. These observations indicated that different vaccination schedules (one or two doses, dosing intervals of 21 days or 6 months for a second dose) may not impact on reactogenicity which is in line with previous experience with AS03 adjuvanted H5N1 vaccine [17].

## Conclusions

The results indicate that flexibility in the dosing interval for AS03_A_ adjuvanted vaccine which could help to reduce the logistic stress on delivery of pandemic vaccination programmes may be possible if a two-dose vaccination policy is required. Potential reduction of antigen content to 1.9 μg HA may also be of interest in the context of potential antigen shortage.

## Competing interests

GSK Biologicals SA was the funding source and was involved in all stages of the study conduct and analysis. GSK Biologicals SA also took charge of all costs associated with the development and publishing of the manuscript. All authors had full access to the data and had the final responsibility to submit the manuscript for publication.

HL, PL, and DD have no conflict of interest to declare. OL, and XD received travel grants from the commercial entity which sponsored the study. AC, PG, and TV were all employees of GlaxoSmithKline group of companies at the time of the manuscript’s development. PG and AC report ownership of stock options.

## Authors’ contributions

All authors participated in the design, implementation, analysis and interpretation of the study. All authors read and approved the final manuscript. AC and PG were involved in all phases of the study, and are part of the clinical team at GSK. TV conducted the data analysis.

## Pre-publication history

The pre-publication history for this paper can be accessed here:

http://www.biomedcentral.com/1471-2334/12/162/prepub

## Supplementary Material

Additional file 1 **Table S1.** Number of subjects enrolled and number of subjects excluded from per-protocol cohort analysis with rationale for exclusion at each time point. Click here for file
