# Supplementary material for: Flexibility of interval between vaccinations with AS03A-adjuvanted influenza A (H1N1) 2009 vaccine in adults aged 18–60 and >60 years: a randomized trial
Source: BMC Infect Dis. 2012 Jul 23;12:162. doi: 10.1186/1471-2334-12-162 (PMC3522029; doi:10.1186/1471-2334-12-162)
Supplement: Additional file 1 — Table S1. Number of subjects enrolled and number of subjects excluded from per-protocol cohort analysis with rationale for exclusion at each time point. [file 1471-2334-12-162-S1.pdf]

Supplementary Table 1: Number of subjects enrolled and number of subjects excluded from per-protocol cohort analysis with rationale for exclusion at each time point

Cohorts (n) /rationale for exclusion	**D0/D21**	**D42**	**D182**	**D203***	**D364**
**Total Cohort**	**313**				
Study vaccine dose not administered	7				
**Total Vaccinated Cohort (TVC)**	**306**	**306**	**306**	**122**	**306**
Administration of vaccine (s) forbidden in the protocol	5	6	27	25	2
Study Vaccine Dose not administered according to protocol	0	1	1	0	1
Noncompliance with vaccination schedule (including wrong and unknown dates)	0	1	1	0	1
Non-compliance with blood sampling schedule (including wrong and unknown dates)	1	5	1	6	8
Essential Serological data Missing	4	8	22	15	33
**Total eliminations from TVC**	**10**	**21**	**52**	**46**	**45**
**per-protocol cohort for immunogenicity (D21/D42)**<br>**per-protocol cohort for persistence (D182/D203*/D364)**	**296**	**285**	**254**	**76**	**261**
**per-protocol cohort for safety**	**301**	**299**	**278**	**97**	**303**

*** GROUP B only**
